# High Prevalence of Childhood Trauma in Australian Adults With Migraine: A Cross‐Sectional Study From a Tertiary Headache Center

**DOI:** 10.1002/brb3.71905

**Published:** 2026-11-05

**Authors:** Lakshini Gunasekera, Shobi Sivathamboo, Eveline Mu, Elspeth Hutton, Helmut Butkueven, Terence O'Brien, Jayashri Kulkarni

**Affiliations:** ^1^ Department of Neurology Alfred Hospital Melbourne Australia; ^2^ Department of Neuroscience, School of Translational Medicine Monash University Melbourne Australia; ^3^ Multidisciplinary Alfred Psychiatry Research Centre, Alfred Health Melbourne Australia; ^4^ HER Centre Australia Department of Psychiatry, School of Translational Medicine Monash University Melbourne VIC Australia; ^5^ Department of Neuroscience, Central Clinical School Monash University Melbourne Victoria Australia; ^6^ Department of Medicine (The Royal Melbourne Hospital) The University of Melbourne Melbourne Victoria Australia; ^7^ Department of Neurology The Royal Melbourne Hospital Melbourne Victoria Australia

**Keywords:** abuse, childhood, deprivation, migraine disorders, neglect, trauma

## Abstract

**Objectives:**

To describe the prevalence of past childhood trauma, including the most common subtypes, in Australian adults with episodic and chronic migraine.

**Background:**

Childhood trauma may alter neurobiological processes that lower pain thresholds and amplify pain signals. International studies show that a history of adverse childhood experiences (ACEs) confers double the risk of migraine development in adulthood, compared to those without a history of childhood trauma. The prevalence of childhood trauma in Australian adults with migraine is unknown.

**Methods:**

Single‐center, cross‐sectional study of adults with a neurologist‐confirmed migraine diagnosis attending a tertiary hospital headache clinic. We invited 1220 eligible patients to complete a validated childhood trauma questionnaire. Responses to the adverse childhood experiences questionnaire, demographic, and clinical data were collected. Significance between binary categorical variables was evaluated using binary logistic regression and reported as odds ratios with 95% confidence intervals.

**Results:**

Of the 514 respondents, most were female (82.7%, 425/514) with mean age 47.1 years (standard deviation [SD] 14.0 years). The proportion of those with episodic migraine (274/514, 53.3%) and chronic migraine (240/514, 46.7%) was roughly equal. Most migraine patients reported at least one traumatic childhood event (419/514, 81.5%). Clinically significant past trauma (defined as ACE scores of ≥ 4) was common (198/514, 38.5%). Past verbal abuse was the most common trauma exposure (247/514, 48.1%). Other trauma exposures were psychiatric diagnosis in parent (223/514, 43.4%), emotional neglect (219/514, 42.6%), physical abuse (182/514, 35.4%), sexual abuse (135/514, 26.3%), parental divorce (195/514, 37.9%), parental substance abuse (145/514, 28.2%), witnessing domestic abuse (108/514, 21.0%), physical neglect (64/514, 12.5%), and parental incarceration (28/514, 5.4%). Higher odds of chronic migraine, but not episodic migraine, were observed with past physical abuse (OR 1.56, *p* = 0.016) and emotional neglect (OR 1.50, *p* = 0.031); however, significance was lost after Bonferroni correction. Deprivational traumas were associated with higher odds of chronic migraine OR 1.56 (*p* = 0.013), whereas threat traumas OR 1.41 (*p* = 0.059) and household dysfunction OR 0.79 (*p* = 0.217) did not show statistical significance.

**Conclusion:**

Australian adults with migraine report high levels of childhood trauma, with verbal abuse being the most common. Understanding the timing, types, severity, and impact of past trauma on the individual may offer further mechanistic insights which could lead to trauma‐based therapies for migraine patients.

## Graphical abstract

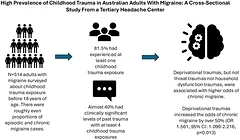

**Abbreviations**
ACE: adverse childhood experiences
HPA axis: hypothalamic‐pituitary‐adrenal axis
ICHD‐3: International Classification of Headache Disorders‐3
OR: odds ratio
PTSD: post‐traumatic stress disorder
SD: standard deviation

## 1 Introduction

There is emerging evidence from large systematic reviews and meta‐analyses that childhood trauma almost doubles the risk of developing migraine (Sikorski et al. 2023; Liu et al. 2024). Further, pooled data from over 100,000 migraine patients have found that childhood trauma predicts both earlier onset of migraine and a more chronic phenotype (Liu et al. 2024). Given that migraine affects roughly 15% of the world's population and contributes to significant personal, societal, and economic burdens (Steiner and Stovner 2023), it is imperative that its causes are investigated at a grassroots level in order to intervene prior to its chronification.

The International Classification of Headache Disorders’ diagnostic criteria for migraine stipulate unilateral onset of throbbing head pain and associated nausea, vomiting, photosensitivity, phonophobia, and head pain on movement (Headache Classification Committee of the International Headache Society 2013). Though there are over 200 different headache disorders (Steiner and Stovner 2023), it is migraine that is the leading cause of disability in the world for people under the age of 50 years (Steiner and Stovner 2023). Given the disproportionate burden of this disease in a relatively young population, the impact of early life stressors such as childhood trauma may play a part in migraine onset and chronification.

Adverse childhood experiences (ACEs) within the family environment include threat traumas (verbal abuse, physical abuse, or sexual abuse); deprivational traumas (emotional or physical neglect); and household dysfunction traumas (parental divorce or separation, witnessing domestic abuse, substance abuse in a parent, psychiatric diagnosis in a parent, or incarceration of a parent) (Mansuri et al. 2020). There is evidence that exposure to ACEs before the age of 18 years, when the brain is still developing, carries a risk of chronic diseases such as migraine and early death (Mansuri et al. 2020; Goodwin et al. 2003; Anda et al. 2010). In fact, every additional ACE accumulated increases the risk of developing ischemic heart disease by 20% when compared to counterparts without any ACEs (Dong et al. 2004). This is significant given migraine with and without aura may be independent risk factors for cardiovascular disease (Bigal et al. 2010). Higher ACE burden also contributes to premature mortality, with those having experienced six or more ACEs living 20 years less than those without any ACEs (Brown et al. 2009). The putative mechanisms of ACE contributing to migraine pathophysiology involve structural changes to brain areas involved in pain perception such as the hypothalamus, prefrontal cortex, and the amygdala; as well as physiological changes to the hypothalamic‐pituitary‐adrenal (HPA) axis with respect to stress hormone secretion (Mansuri et al. 2020; Tietjen and Peterlin 2011; Siego et al. 2021; Sic et al. 2025). Exposure to childhood maltreatment has been associated with structural changes to key brain areas including the hippocampus, amygdala, frontal cortex, and corpus callosum which are all brain areas involved in pain perception (Tietjen and Peterlin 2011; Siego et al. 2021; Sic et al. 2025; Teicher and Samson 2016). Different traumatic events may also preferentially impact different brain areas; for example, threat traumas can lead to atrophy of the hippocampus, amygdala, and prefrontal cortex while deprivational traumas can reduce the thickness and volume of the association cortex (Sikorski et al. 2023; Teicher and Samson 2016). The current literature supports corpus callosum atrophy in response to trauma (Tietjen and Peterlin 2011; Siego et al. 2021; Sic et al. 2025; Teicher and Samson 2016). While the exact downstream effects of these changes are unclear, it is conceivable that they may lead to altered pain perception responses via a final common pathway.

In Australia, childhood trauma is highly prevalent within the population, with studies showing that up to 62% of adults have experienced at least one form of childhood maltreatment (Higgins et al. 2023; Mathews et al. 2023). Cumulative trauma exposure is common, with 39% of the aforementioned population endorsing multiple childhood trauma exposures (Higgins et al. 2023).

We lack Australian data looking at the prevalence and subtypes of childhood trauma in adults with migraine, which may inform risk mitigation and prevention strategies. We hypothesize that the prevalence of childhood trauma would be higher in a population of patients with migraine who attend a tertiary headache clinic within Melbourne, Australia, when compared to the Australian population prevalence.

## 2 Methods

### 2.1 Study Design and Setting

This was a single center, cross‐sectional, survey‐based study describing the prevalence and subtypes of past childhood trauma in adults with migraine.

### 2.2 Setting

This study was conducted at the Alfred Hospital Headache clinic in Melbourne, Australia. This clinic sits within a tertiary hospital and sees approximately 800 patients per year for headache treatment. This multidisciplinary center includes headache neurologists, a headache nurse, a headache psychologist, and headache dietitian. Eligible patients were considered for study inclusion between January 1, 2024 and June 30, 2025. No follow‐up occurred after study completion.

### 2.3 Participants

Adults > 18 years attending the Alfred Hospital Headache clinic were offered this study if there was a neurologist‐confirmed diagnosis of migraine per the International Classification of Headache Disorders (ICHD‐3) (Headache Classification Committee of the International Headache Society 2013). Non‐migraine primary headaches and non‐English‐speaking patients were excluded. Verbal consent was obtained and further confirmed by voluntary survey completion.

### 2.4 Variables and Data Sources

For all eligible patients, the survey was offered at the conclusion of their clinic appointment or sent to their listed email address using a secure REDCap link. Patients were able to stop the surveys or withdraw from the study if they became distressed. Mental health consultations were available for distressed individuals.

Variables collected were age, sex assigned at birth, baseline headache characteristics, and responses to the Adverse Childhood Experiences‐Questionnaire (ACE‐Q) (Felitti et al. 1998). This survey takes 1–2 min to complete and has been designed to be self‐reported (Felitti et al. 1998).

The specific headache characteristics collected were episodic or chronic migraine label per consultant neurologist and self‐reported monthly headache days (MHD), monthly migraine days (MMD), and monthly crystal‐clear days.

The ACE questionnaire has been shown to have good internal consistency and criterion validity (Loncar et al. 2026). In addition, many studies have found a relationship between early life adversity (using the ACE questionnaire) and later‐life adverse health outcomes (Mathews et al. 2023). The ACEs are thus used in epidemiological studies to find associations between trauma during neurodevelopment and medical comorbidities in later life.

The specific ACEs we enquired about were childhood verbal abuse (including swearing, insults, put‐downs, humiliating comments, or verbal threats), physical abuse (being pushed, grabbed, slapped, or hit), sexual abuse (being sexually touched or fondled, trying to have or actually having oral, anal, or vaginal penetration), emotional neglect (including not feeling loved, valued, or included), physical neglect (lacking food, clean clothes, or medical care), witnessing domestic violence, living with a parent who suffered from a mental health diagnosis (including a parent who attempted suicide) living with a parent who suffered from a substance abuse problem (including alcohol or street drugs), parental separation, or having a parent who was incarcerated.

### 2.5 Bias

This study was offered to every individual with a migraine diagnosis during the study period, thus minimizing selection bias since every eligible patient was able to participate. Patients were advised that their involvement or non‐involvement in the study would not impact their clinical care. Reporting bias is expected to be low because data collection was anonymous, thus allowing for honest reporting without altering responses to suit clinician, researcher, or societal expectations. Together, these features allow for a representative sample of adults with migraine. However, given the survey contents about childhood events, the risk of recall bias could not be reasonably mitigated. No reminder emails were sent to patients who did not complete the survey, thus minimizing the risk of coercive involvement. Patients were also not approached a second time to partake in the study if they ever indicated they did not wish to participate.

### 2.6 Sample Size

An a priori sample size calculation was conducted using a one‐sample test of proportions, comparing the Australian population prevalence of childhood trauma with that expected of migraine patients in a hospital cohort. The estimated Australian population prevalence of childhood trauma is 62% (Higgins et al. 2023), and we hypothesized that our hospital cohort has a higher prevalence of childhood trauma. Assuming a null hypothesis of 0.62, a one‐sided alternative hypothesis of > 0.62, an expected hospital prevalence of 0.70, an alpha of 0.05, and a power of 0.80, the required sample size was 370 patients.

We opted for a one‐sided test of proportions since a trauma prevalence less than 62% in this cohort would not be clinically meaningful. The expected prevalence of 70% was chosen to ensure small differences in prevalence were detected (i.e., 8%), while still being higher than the population norm. Accounting for a 20% lack of survey completion, we needed a sample size of 400 participants to show this difference.

### 2.7 Statistical Analysis

Categorical variables were reported as counts and percentages. Continuous variables were reported as the sample mean with standard deviations. Associations between predictors and binary outcomes were assessed using binary logistic regression, and the results were reported as odds ratios with 95% confidence intervals, and *p*‐values with the significance level set at *p* < 0.05.

## 3 Results

We invited 1220 eligible patients to partake in our study (male: 218/1220, 17.9%; female: 1002/1220, 82.1%). The response rate was 42.1% (514/1220). All 514 complete responses were included in the statistical analysis.

Of the 514 respondents, most were female (82.7%, 425/514). Men (85/514, 16.5%) and other gender identities (4/514, 0.8%) made up a minority. The mean age of respondents was 47.1 years (SD 14.0 years). The proportion of episodic migraine (274/514, 53.3%) and chronic migraine (240/514, 46.7%) was roughly equal. When comparing the migraine burden in episodic and chronic migraine subpopulations, respectively, we noted a difference in MHD (10.5 vs. 20.1 days, *p* < 0.001), MMD (3.4 vs. 17.8 days, *p* < 0.001), and crystal‐clear days (15.1 vs. 6.1 days, *p* < 0.001). In the cohort (*n* = 514), the mean MHD was 15.0 days, the mean MMD was 10.1 days, and the mean crystal‐clear days were 10.9 days.

The prevalence of previous childhood trauma in our clinic population was 81.5% (*n* = 419/514, 95% CI: 78.1%–85.0%) which was significantly higher than the general Australian population prevalence of 62% (one‐sample exact binomial test, *p* < 0.001).

Our observed 81% childhood trauma prevalence in migraine patients is also higher than the expected 70% which was used as a conservative estimate in our power calculation. The breakdown of childhood trauma in our population is as follows: zero traumatic childhood events (95/514, 18.3%), 1 event (83/514, 16.2%), 2 events (78/514, 15.2%), 3 events (61/514, 11.9%), 4 events (52/514, 10.1%), 5 events (57/514, 11.1%), 6 events (38/514, 7.4%), 7 events (24/514, 4.8%), 8 events (9/514, 1.8%), 9 events (17/514, 3.3%), and 10 events (1/514, 0.0%). Clinically significant levels of childhood trauma (defined as an ACE score ≥ 4) were reported in 38.5% of our cohort (198/514). The overall number of ACE exposures in our cohort is seen in Figure 1.

**FIGURE 1:**
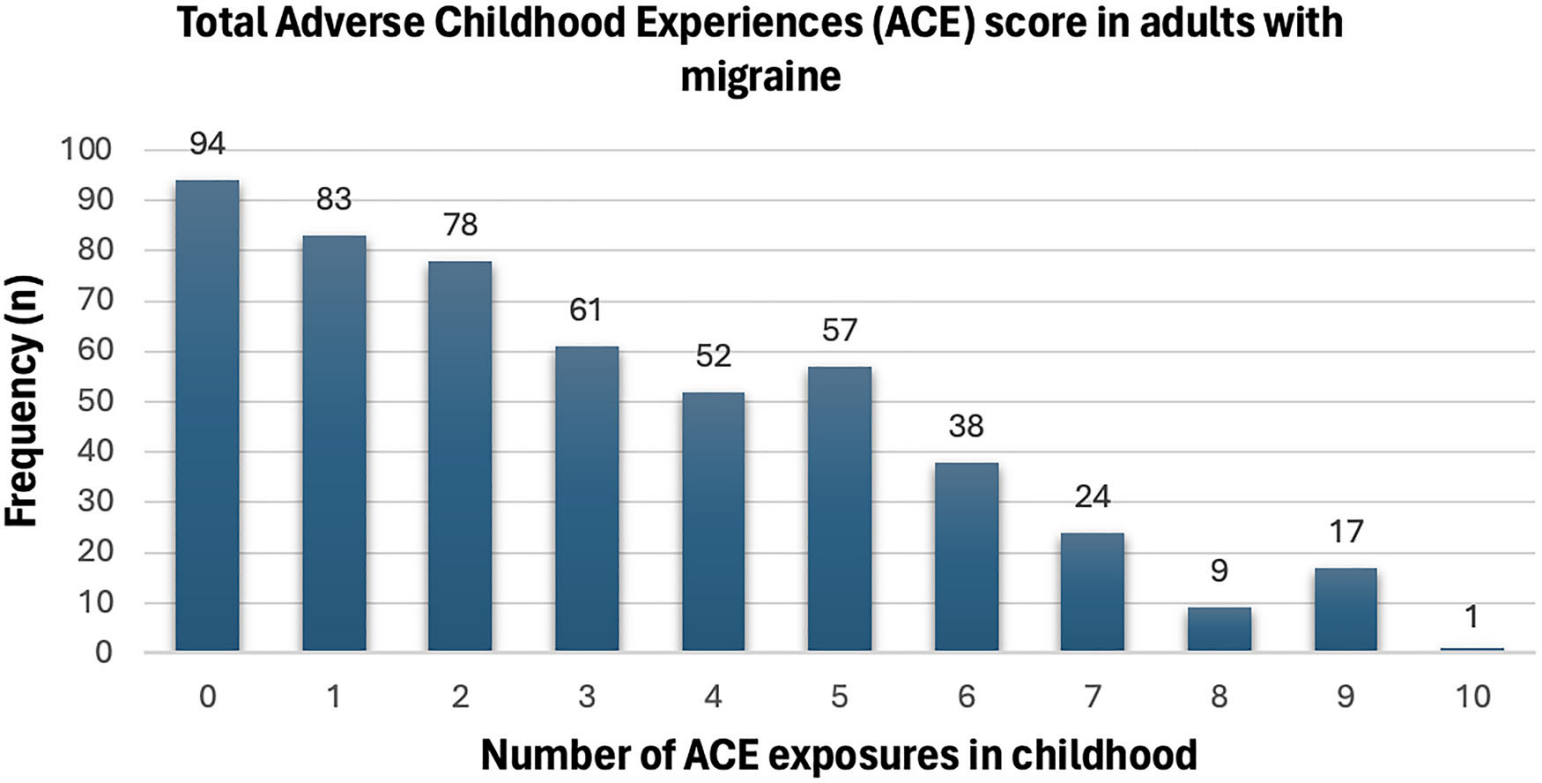
Total adverse childhood experiences (ACE) scores in adults with migraine.

Reported traumatic exposures, in order of decreasing prevalence, were: verbal abuse (247/514, 48.1%), parental psychiatric diagnosis (223/514, 43.4%), emotional neglect (219/514, 42.6%), physical abuse (182/514, 35.4%), sexual abuse (135/514, 26.3%), parental divorce (195/514, 37.9%), parental substance abuse (145/514, 28.2%), witnessing domestic abuse (108/514, 21.0%), physical neglect (64/514, 12.5%), and parental incarceration (28/514, 5.4%). The overall ACE subdomains are summarized in Figure 2.

**FIGURE 2:**
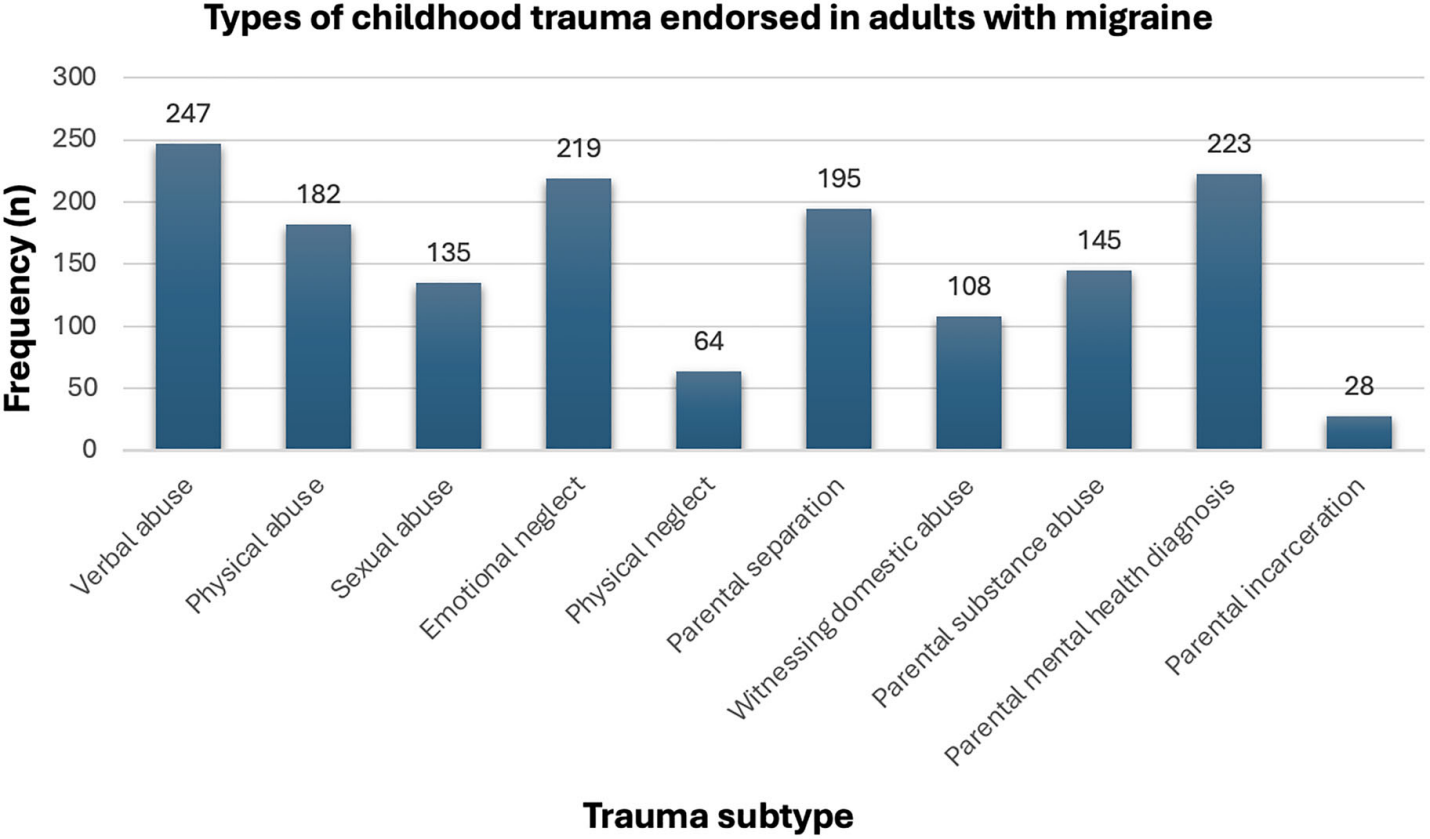
Types of childhood trauma endorsed in adults with migraine.

Table 1 shows the breadth of different trauma exposures reported in those with clinically significant past trauma (ACE scores ≥ 4) compared to those with relatively fewer trauma exposures (ACE < 4). The total ACE score is calculated by adding individual trauma exposures, so it is expected that higher ACE scores reflect endorsing higher numbers of trauma types. The intention of this table is to show that participants with ACE ≥ 4 report exposures across all ACE domains, consistent with the multidomain nature of cumulative adversity. There is no single trauma exposure that differentiates those with relatively high trauma scores overall.

**TABLE 1:** Differences between clinically significant ACE scores and reported childhood trauma subtypes.

Type of trauma	ACE score ≥ 4 (*n* = 198)	ACE score < 4 (*n* = 316)	Odds ratio (95% confidence interval)	*p* value
Verbal abuse	180	67	37.164 (21.345–64.708)	< 0.001
Physical abuse	144	38	19.509 (12.301–30.941)	< 0.001
Sexual abuse	94	41	6.062 (3.941–9.326)	< 0.001
Emotional neglect	156	63	2.476 (1.848–3.318)	< 0.001
Physical neglect	62	2	71.574 (17.259–296.814)	< 0.001
Parental separation or divorce	120	75	4.944 (3.363–7.267)	< 0.001
Witnessed domestic abuse	95	13	21.497 (11.550–40.011)	< 0.001
Parental substance abuse	109	36	9.526 (6.099–14.878)	< 0.001
Parent with mental health diagnosis	145	78	8.348 (5.565–12.521)	< 0.001
Parental incarceration	19	9	3.621(1.604–8.174)	< 0.001

*Note*: Differences between clinically significant ACE scores and types of previous abuse experience.

Exploratory analyses were conducted to see if there were any individual ACE items that correlated with migraine chronicity or sex differences between trauma types. Findings of these exploratory analyses are shown in Tables 2 and 3 respectively. Chronic migraine, but not episodic migraine, was associated with previous physical abuse (odds ratio [OR] 1.561, 95% CI: 1.085–2.246, *p* = 0.016) and previous emotional neglect (OR 1.495, 95% CI: 1.052–2.125, *p* = 0.025). Both exposures increased the odds of adult migraine by at least 50%. However, significance was lost after Bonferroni corrections. Similarly, sex‐specific analyses showed that verbal abuse was more common in male migraine patients, while living with a parent suffering from a mental health disorder was more common in female migraine patients; both lost significance after Bonferroni corrections.

**TABLE 2:** Differences between migraine frequency and types of childhood abuse.

Type of trauma	Episodic migraine (*n* = 274)	Chronic migraine (*n* = 240)	Odds ratio (95% confidence interval)	*p* value
Verbal abuse	124	123	1.272 (0.899–1.800)	NS (0.175)
Physical abuse	84	98	1.561 (1.085–2.246)	0.016^a^
Sexual abuse	64	71	1.379 (0.930–2.044)	NS (0.110)
Emotional neglect	104	115	1.495 (1.052, 2.125)	0.025^a^
Physical neglect	27	37	1.677 (0.982–2.832)	NS (0.059)
Parental separation or divorce	103	92	1.032 (0.722–1.475)	NS (0.863)
Witnessed domestic abuse	51	57	1.362 (0.890–2.084)	NS (0.155)
Parental substance abuse	80	65	1.110 (0.755–1.633)	NS (0.595)
Parent with mental health diagnosis	120	103	1.036 (0.731–1.470)	NS (0.841)
Parental incarceration	19	9	1.912 (0.848–4.311)	NS (0.118)

*Note*: Differences between migraine frequency and types of previous abuse experienced.

^a^ When Bonferroni corrections were applied across each trauma category to account for multiple testing, there were no associations between any of the trauma categories and presence of migraine chronicity with the adjusted significance threshold (0.05/10 exposures, α threshold = 0.005).

**TABLE 3:** Sex differences in childhood trauma exposures.

Type of trauma	Male patients (*n* = 85)	Female patients (*n* = 425)	Odds ratio (95% confidence interval)	*p* value
Verbal abuse	46	199	0.480 (0.247–0.933)	0.031^a^
Physical abuse	30	149	1.291 (0.677–2.462)	0.437
Sexual abuse	17	117	1.803 (0.964–3.371)	0.065
Emotional neglect	32	185	1.591 (0.858–2.949)	0.140
Physical neglect	13	51	0.639 (0.285–1.430)	0.276
Parental separation or divorce	33	161	0.935 (0.548–1.593)	0.804
Witnessed domestic abuse	24	84	0.563 (0.297–1.069)	0.079
Parental substance abuse	26	119	0.901 (0.500–1.623)	0.729
Parent with mental health diagnosis	29	192	1.995 (1.139–3.494)	0.016^a^
Parental incarceration	4	24	1.401 (0.447–4.395)	0.563

*Note*: Differences between childhood trauma exposures in male versus female patients.

^a^ When Bonferroni corrections were applied to account for multiple testing none of the exposures were significantly different between males and females in our sample (0.05/10 exposures, adjusted alpha threshold of 0.005).

Further classifying trauma exposures into threat traumas, deprivational traumas, and household dysfunction showed possible differing risk profiles for chronic migraine. Deprivational traumas were associated with higher odds of chronic migraine (OR 1.561, 95% CI: 1.099–2.216, *p* = 0.013), whereas this trend was not observed in those reporting threat traumas (OR 1.407, 95% CI: 0.987–2.005) (*p* = 0.059) or household dysfunction (OR 0.792, 95% CI: 0.548–1.146) (*p* = 0.217). The differential impact of different traumatic exposures is seen in Figure 3. Deprivational traumas continued to show significance in increasing the odds of migraine after Bonferroni corrections. There was no difference in migraine chronicity with respect to sex (*p* = 0.171) or mean age (*p* = 0.594).

**FIGURE 3:**
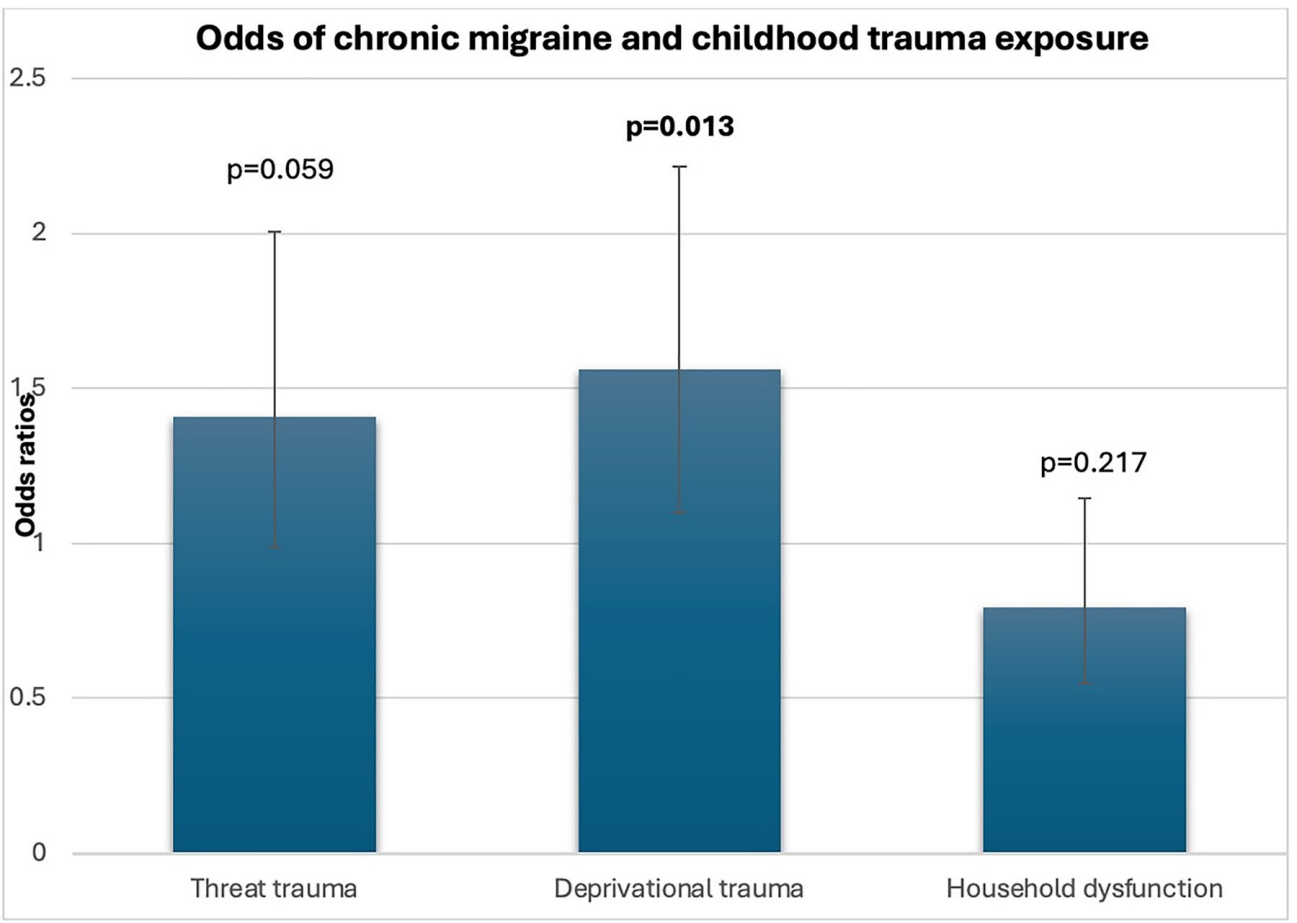
Odds of adult chronic migraine diagnosis based on childhood trauma exposure subtype. After Bonferroni correction, (0.05/3 = adjusted alpha threshold 0.017), deprivational traumas continued to show significance.

Figure 3 examines three potential childhood trauma categories which may increase the odds of chronic migraine. Table 2 examines which, if any, of the 10 ACE trauma exposures may increase the odds of chronic migraine. As the latter analysis is a more detailed and granular analysis of the first one, we calculated separate alpha thresholds for these two hierarchical analyses, rather than condensing them into one analysis.

There was no significant difference between clinically significant ACE scores and migraine frequency (*p* = 0.313). There were no statistically significant differences between episodic migraine and chronic migraine with respect to total ACE scores (*p* = 0.562) or mean ACE scores (*p* = 0.129). There was also no difference between mean ACE scores based on sex (*p* = 0.263). Though it did not reach statistical significance, there was a trend toward clinically significant levels of past trauma in younger patients (mean age 45.6 years vs. 48.0 years, *p* = 0.055).

## 4 Discussion

Australian population‐based studies reveal that childhood maltreatment is common (Higgins et al. 2023; Mathews et al. 2023; Mathews et al. 2023; Moore et al. 2015). The first study regarding child maltreatment in Australia was a systematic review and meta‐analysis showing that the pooled prevalence of childhood trauma ranged from 2% (childhood neglect) to 9% (for each of physical abuse, sexual abuse, and emotional abuse) (Moore et al. 2015). However, this is likely to have been a significant underestimate given the reliance on figures derived from mandatory reporting, which required cases severe enough to warrant reporting and also required substantiation of claims. Since then, the Australian Child Maltreatment Study (ACMS) has shown that 62% of Australian adults had experienced childhood maltreatment, with 40% of these adults having exposure to multiple trauma types. In this study of five maltreatment exposures, they found past physical abuse in 32%, sexual abuse in 28.5%, emotional abuse in 30.9%, neglect in 8.9%, and exposure to domestic violence in 39.6%.

This study describes the prevalence of, and the most common subtypes of, childhood trauma in adults with migraine in Australia. Our study had four key findings. First, there was a significant overlap between migraine and childhood trauma, with over 80% of migraine patients reporting childhood trauma. Second, clinically significant levels of past trauma, with an ACE score ≥ 4, occurred in 40% of people, showing that traumatic exposures tend to accumulate in vulnerable individuals. Third, deprivational traumas were associated with higher odds of chronic migraine (*p* = 0.013) while threat traumas and household dysfunction traumas were not statistically significant for the same. Fourth, both physical abuse (*p* = 0.016) and emotional neglect (*p* = 0.031) were associated with higher odds of chronic migraine, though significance was lost after Bonferroni correction.

We have found a higher prevalence of childhood trauma in our study population of migraine patients than in the Australian population‐based data (81.5% vs. 62%, one‐sample exact binomial test, *p* < 0.001). This raises the question about whether childhood trauma could be investigated as a potential risk factor for the development of migraine. Though we cannot directly compare our results with the general population (since this is expected to contain a mixture of people both with and without migraine) and our results do not imply causation, our present study does raise interesting questions for future prospective studies with larger samples and a control population without migraine. If an association is established between migraine and childhood trauma, we posit that trauma‐based therapies may be a useful adjunct in migraine care.

Our study, showing that over 80% of sampled migraine patients had previous childhood trauma, reports higher levels of childhood trauma than studies from other tertiary headache clinics, which reported 10%–60% previous maltreatment (Tietjen et al. 2010a; Tietjen et al. 2010b; Tietjen et al. 2007). We found that verbal abuse was the most common form of trauma, in contrast to other studies where physical and sexual abuse were commonest (Tietjen et al. 2010a; Tietjen et al. 2010b; Tietjen et al. 2007). This distinction is important since not all trauma types have an equivalent impact on neurodevelopment and headache onset (Sikorski et al. 2023). While the ACE questionnaire gives equal weighting to all items, there is emerging evidence that threat traumas and deprivational traumas affect different areas of the brain during development (Sikorski et al. 2023; Tietjen et al. 2017; Golding 1999; Reuchlein et al. 2016). Our study has supported these theories in exploratory analyses showing that deprivational traumas were associated with higher odds of migraine chronicity, while threat and household dysfunction traumas were not statistically significant for this association. Future studies with larger samples would be required to further explore these hypothesis‐generating exploratory analyses.

Similar to Australian population‐based studies showing that trauma exposures tend to be cumulative within an individual (Higgins et al. 2023), our study has shown the same trend in Table 1 where those with clinically significant levels of trauma endorse multidomain traumatic exposures rather than concentration within a single trauma type.

Though the aim of our study was not to evaluate the association between chronic migraine and specific trauma types or sex differences, exploratory analyses were conducted with the aim of generating hypotheses for future studies. Binary logistic regression showed that physical abuse and emotional neglect increased the odds of chronic migraine, but both lost significance after Bonferroni corrections were applied to account for multiple comparisons. Similarly, sex‐stratified trauma exposures showed that childhood verbal abuse was more common in male migraine patients, while a parental mental health diagnosis was more common in female patients with migraine. However, neither of these exposures retained significance after Bonferroni corrections. Loss of significance after accounting for multiple comparisons could be due to a lack of power to detect subtle differences given our modest sample size or an absence of an effect. Sex‐specific analyses may be underpowered to detect subtle differences between male and female participants, given the relatively smaller number of male participants in this sample.

Interestingly, the Bonferroni correction continued to show that deprivational traumas were associated with higher odds of chronic migraine, whereas threat traumas and household dysfunction did not display similar statistical significance. Future prospective studies with larger samples could be useful to thus evaluate subtle differences in trauma subtypes or sex‐differences, which may be lost herein due to a potentially underpowered study.

There is ample evidence from the literature that childhood trauma and migraine headaches commonly co‐occur (Sikorski et al. 2023; Liu et al. 2024; Mansuri et al. 2020; Goodwin et al. 2003; Anda et al. 2010; Tietjen and Peterlin 2011; Siego et al. 2021; Tietjen et al. 2010a; Tietjen et al. 2010b; Tietjen et al. 2007; Tietjen et al. 2017; Golding 1999; Reuchlein et al. 2016; Tietjen et al. 2010c; Peterlin et al. 2008; Tietjen et al. 2015; Hammond et al. 2022; Kucukgoncu et al. 2014; Bottiroli et al. 2025; Baiden et al. 2021; Genizi et al. 2014; Fuller‐Thomson et al. 2010; Gelaye et al. 2016; Stensland et al. 2014; Fuh et al. 2010; Brennenstuhl and Fuller‐Thomson 2015; Mays et al. 2022; Santos et al. 2014). Existing studies, however, have limitations which make direct comparison between studies difficult and pose a challenge to understanding the exact associations. The definition of “child” varies widely with 15 years, 16 years, and 18 years all being included in the childhood maltreatment literature (Moore et al. 2015). Some studies underestimate the prevalence of childhood trauma by involving only adolescent participants who are unable to comment on events through all eighteen years of their lives (Hammond et al. 2022; Genizi et al. 2014; Fuh et al. 2010). Other studies rely on parental reports of abuse, which is prone to reporting bias, especially if the parent is a perpetrator of abuse (Mansuri et al. 2020; Tietjen and Peterlin 2011; Hammond et al. 2022; Baiden et al. 2021). In that regard, self‐reported past abuse may be more reliable; however, limiting questions to emotional abuse and sexual abuse alone reduces understanding of the full abuse spectrum impacting migraine (Tietjen et al. 2015). Similarly, another study which only looked at the impact of physical abuse on migraine development ignored the very real impact of deprivational trauma on pain perception and escalation (Fuh et al. 2010). Combining both headache and migraine together as one outcome of interest is problematic since migraine involves a specific pathophysiology which may not overlap with other primary headache disorders (Mansuri et al. 2020; Kucukgoncu et al. 2014; Stensland et al. 2014). Conversely, using only a self‐reported migraine diagnosis may reduce the reliability of results, since the diagnosis has not been verified by a neurologist (Brennenstuhl and Fuller‐Thomson 2015).

Despite the limitations in how the bidirectional nature of trauma and migraine is captured, their negative impacts are well described. Childhood trauma is associated with earlier onset migraine, and its conversion from an episodic phenotype to a chronic phenotype (Liu et al. 2024; Tietjen et al. 2010c; Bottiroli et al. 2025; Mays et al. 2022). More broadly, childhood trauma is linked to general ill health, including mental health disorders, such as post‐traumatic stress disorder, which can intensify migraine (Felitti et al. 1998; Peterlin et al. 2008; Fuller‐Thomson et al. 2010; Santos et al. 2014). This means that trauma both directly and indirectly impacts migraine, thus its treatment could have numerous benefits, even for comorbidities. As it relates to the administered ACE‐Q, each additional ACE accumulated increases the risk of cardiovascular disease by 20% (Dong et al. 2004), while six or more ACEs reduced life expectancy by 20 years when compared to counterparts without ACEs (Hughes et al. 2017). Migraine with and without aura are both independent risk factors for cardiovascular disease (Bigal et al. 2010), thus traumatic childhood exposures become even more important to avoid, as they are preventable causes of additive risk in this population.

We speculate that a key reason for trauma predisposing to migraine could be due to HPA axis dysregulation after exposure to stress (Mansuri et al. 2020). Normally, hypothalamic secretion of corticotropin releasing hormone (CRH) leads to secretion of adrenocorticotropic hormone (ACTH) by the pituitary gland, which then causes cortisol secretion by the adrenal gland. Acutely during stress this can be advantageous by activating the sympathetic nervous system to respond to the acute stressor (Mansuri et al. 2020). Chronic stress may have different effects. Studies show that cortisol can be chronically high in patients with chronic migraine with underlying chronic stress (Tietjen and Peterlin 2011), while other studies show that women with significant sexual abuse histories chronically have low cortisol levels (Trickett et al. 2011). The unpredictable cortisol levels may be mediated by peripheral cortisol resistance, glucocorticoid receptor downregulation, and overall blunted responses to cortisol binding due to HPA axis dysregulation and epigenetics (Anda et al. 2010; Tietjen and Peterlin 2011). Regardless of whether cortisol levels are chronically low or high, their dysregulation could have significant effects on migraine onset and persistence (Sic et al. 2025). For example, chronically high cortisol is associated with hippocampal atrophy and increased neuroinflammation, which may alter pain perception in migraine patients (Sic et al. 2025). Since the hippocampus normally regulates the HPA axis, its atrophy could mean that the chronic stress response may not be turned off or down in those with frequent headaches (Tietjen and Peterlin 2011). In contrast, cortisol normally suppresses excess inflammation, so if it is low, more inflammation could occur (Mansuri et al. 2020). This uncontrolled sterile chronic inflammation may further sensitize the trigeminal nerve leading to further release of inflammatory cytokines and neuropeptides, which could be implicated in migraine pathophysiology by sustaining the inflammatory milieu (Sic et al. 2025). Furthermore, chronic sympathetic system activation can increase muscle tension, alter sleep patterns, and alter metabolism—all of which can be implicated in triggering or maintaining migraine (Sic et al. 2025).

Our study has several strengths. We confirm that migraine patients commonly report high levels of childhood trauma. Our population had neurologist‐confirmed migraine, thus excluding other primary headache disorders with different pathogenetic mechanisms. We also used self‐reported ACE scores, which allow us to evaluate the impact of trauma on the individual, by the individual. In contrast, other studies asked for traumatic childhood events to be reported by parents on behalf of the child, which has the tendency to underreport findings, especially if a parent is the perpetrator (Mansuri et al. 2020; Tietjen and Peterlin 2011; Hammond et al. 2022; Baiden et al. 2021). Though self‐reporting past trauma may be prone to recall bias, our sample size of over 500 respondents ensures that under‐ or over‐reporting trauma is absorbed by the large study size, which far exceeds our sample size calculation.

This study has several limitations. First, retrospective recall of answers may introduce recall bias. However, there is evidence that trauma‐related dissociation and depersonalization often lead to underreporting of events, not fabrication of events (Carlson and Dalenberg 2000). Second, we lack information about the severity or timing of the trauma with respect to migraine onset and critical stages in neurodevelopment. It would be useful for future studies to evaluate whether physical abuse specifically included head trauma or concussion, and its relative onset with respect to migraine attacks. If head trauma were present, the differences in repeated head trauma versus an isolated head injury would offer insights into the mechanisms of migraine pathogenesis. The impact of emotional neglect would be important to explore with respect to an individual's support systems, attachment style, and access to psychological support. Third, this study is limited by content pertaining to trauma within the family unit. We received qualitative feedback that participants would have liked questions relating to natural disasters, medical illnesses, iatrogenic trauma, peer bullying, and motor vehicle accidents. While we cannot realistically cover all potential traumatic exposures, we anticipate that the family unit is the best focus for childhood‐related trauma studies. Fourth, given the sensitive nature of the questions, investigators aimed to minimize patient distress by anonymous reporting and minimal identifiable medical information; however, confounders such as depression leading to underreporting cannot be ascertained. The large sample size and stark difference in prevalence when compared to Australian population data is expected to buffer these confounding effects. Fifth, the response rate to our survey invitation was 40%. Of these, 87% were female, which is higher than the 82% female proportion in the total 1220 eligible participants. These results may thus not be representative of the entire cohort. We did not collect reasons for non‐participation, so it is difficult to comment on whether patients with trauma histories were more likely to respond. Finally, some of the observed associations lost significance after Bonferroni correction, such as the higher odds of chronic migraine with past physical abuse and emotional neglect, and we cannot reliably differentiate between an absence of a true effect, an underpowered study, or both.

Larger studies with a control group are required to confirm hypotheses posited in this study. Future studies could include different trauma types, timing, attachment styles, self‐perceived availability of support, and coping styles. Future studies could also look at ACE burden and migraine prevalence in particularly high‐risk groups, such as Aboriginal and Torres Strait Islander populations. With further research, trauma‐based therapies for migraine management may be useful in these cohorts.

## Author Contributions

L.G. and S.S. conceived of the study. L.G., S.S., E.H., and J.K. were involved in the study design. L.G. was involved in ethics acquisition, data collection, statistical analysis, preparation of the first draft of the manuscript, and all the subsequent revisions. All the other authors contributed equally to project oversight and manuscript revision.

## Funding

The authors have nothing to report.

## Ethics Statement

The study was approved by the Alfred Hospital Human Research Ethics Committee (Local ref. 280/25). Patients completed electronic consent prior to survey initiation.

## Conflicts of Interest

Authors have no relevant conflicts of interest, but general disclosures are as follows: Lakshini Gunasekera has received funding from the Victorian Government catalyst grant program to investigate hormonal therapies for menstrual migraine, writer's honoraria from Pain Management Today, and speaker honoraria from Lundbeck and the Victorian Government Department of Transport and Planning.

Shobi Sivathamboo is the recipient of a National Health and Medical Research Council (NHMRC) Investigator Award (2025610). She is supported by Research Program Grants from the National Institute of Health (NIH) (1U54AT012307‐01 and 1R01NS123928‐01). She reports salary support paid to her institution from Jazz Pharmaceuticals and Quadlytics for clinical‐trial‐related activities; she receives no personal income for these activities.

Eveline Mu has received funding from the Department of Health (VIC), the Norman Beischer Medical Research Foundation, the Hugh D.T. Williamson Foundation, the Monash University, the Wolinski Foundation, the Grummit Family, the One in Five Association, the Australian Communities Foundation, the Women's Health Research Translation and Impact Network, and the Li Family Trust.

Terence O'Brien's institution has received research funding from Eisai, UCB Pharma, LivaNova, ES Therapeutics, Jazz Pharmaceuticals, and Kinoxis Therapeutics. Terence O'Brien was funded by NHMRC Investigator Grants (Nos. APP1176426 and APP2034258). He has also received competitive grant funding from the NHMRC, MRFF, NINDS, and the DoD.

Helmut Butkueven has received institutional (Monash University) funding from NHMRC (L1 Investigator Grant), MRFF Australia, MSAustralia, the Trish Foundation, the Pennycook Foundation, Biogen, F. Hoffmann‐La Roche Ltd, Merck, Alexion, CSL, UCB, and Novartis; carried out contracted research for Novartis, Merck, F. Hoffmann‐La Roche Ltd, and Biogen; taken part in speakers’ bureaus for Biogen, UCB, Novartis, F. Hoffmann‐La Roche Ltd & Merck (honoraria paid to institution), and has received conference support from Merck and Novartis.

Elspeth Hutton has served on advisory boards for Sanofi‐Genzyme, Novartis, Teva, Eli Lilly, Allergan, Lundbeck, been involved in clinical trials sponsored by Novartis, Teva, Xalud, Cerecin, and has received payment for educational presentations from Allergan, Teva, Eli Lilly, and Novartis.

Jayashri Kulkarni has received grant support for research from the Stanley Medical Research Institute (Washington, USA), the Norman Beischer Foundation, the National Health and Medical Research Council of Australia, AstraZeneca, Mayne Pharma, Servier, Eli Lilly, Jansen‐Cilag, Neurosciences Australia, and the Department of Human Services (Victoria, Australia). She has received honoraria as a speaker for Jansen‐Cilag, Lundbeck, AstraZeneca, and Bristol Myers Squibb, and is an advisory board member for Janssen‐Cilag, Lundbeck, Roche, and Pfizer.

## Data Availability

Qualified investigators seeking access to anonymized patient‐level data for the purpose of replicating the study's procedures and findings may do so upon request. Data sharing will be subject to obtaining the necessary local ethics approval prior to release.
